# Stuck in the Sump: A Rare Complication of Biliary Surgery

**DOI:** 10.7759/cureus.75090

**Published:** 2024-12-04

**Authors:** Daniel F Wagner, Leanne Sowunmi, Ross Driscoll

**Affiliations:** 1 Internal Medicine, Western Michigan University Homer Stryker M.D. School of Medicine, Kalamazoo, USA

**Keywords:** biliary complication, case report, cholangitis, cholecystectomy, choledochoduodenostomy, mrcp, sump syndrome

## Abstract

Sump syndrome is a rare complication of biliary surgery that is now rarely seen in the era of Endoscopic Retrograde Cholangiopancreatography (ERCP). It occurs when the distal common bile duct becomes obstructed between an anastomosis from a choledochoduodenostomy (CDD) and the ampulla of Vater, forming a sump that accumulates debris. Sump syndrome should be considered as a diagnosis in patients who present with cholangitis or pancreatitis and any history of biliary diversion, regardless of the time of presentation. We report a 61-year-old female who presented with sump syndrome two years after undergoing a side-to-side CDD.

## Introduction

Sump syndrome is a rare complication of biliary surgery, mainly choledochoduodenostomy (CDD) or choledochojejunostomy, that is now rarely seen in the Endoscopic Retrograde Cholangiopancreatography (ERCP) era, with a reported prevalence of between 0 - 9.6% [1,2]. The CDD procedure may be performed when the common bile duct (CBD) drains improperly and has many indications, including strictures or obstruction of the bile duct and pancreas, biliary fistulas, recurrent bile duct stones, stenosis of the sphincter of Oddi, and choledochal cysts [2,3].

Classically, in sump syndrome, the distal bile duct becomes obstructed after CDD, with the CBD segment between the anastomosis and the ampulla of Vater becoming a reservoir or sump. This likely occurs due to low filling pressure in the distal bile duct because the well-functioning anastomosis interferes with normal distal peristalsis and drainage. When debris accumulates in the sump, recurrent pain episodes, fever, cholangitis, pancreatitis, biliary obstruction, or hepatic abscesses may develop [4].

We present the case of a 61-year-old female patient who exhibited clinical signs concerning for ascending cholangitis. Her detailed medical history, imaging studies, and ERCP led to the diagnosis of sump syndrome. This case was previously presented as a meeting abstract at the 2024 Michigan Chapter of the American College of Physicians Annual Scientific Meeting on October 26, 2024.

## Case presentation

**Figure 1:**
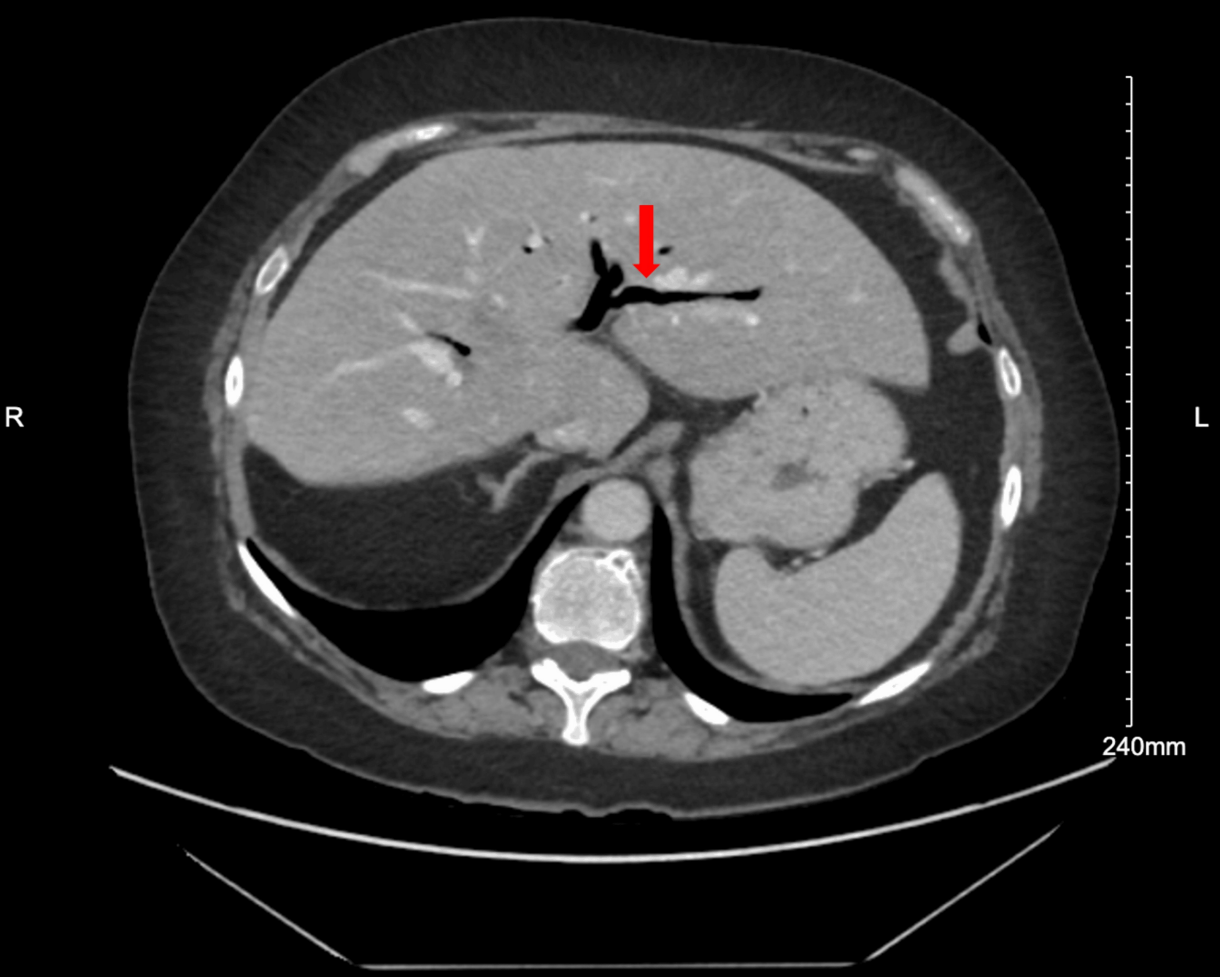
Axial computed tomography (CT) abdomen/pelvis with contrast. Arrow points to the pneumobilia.

**Figure 2:**
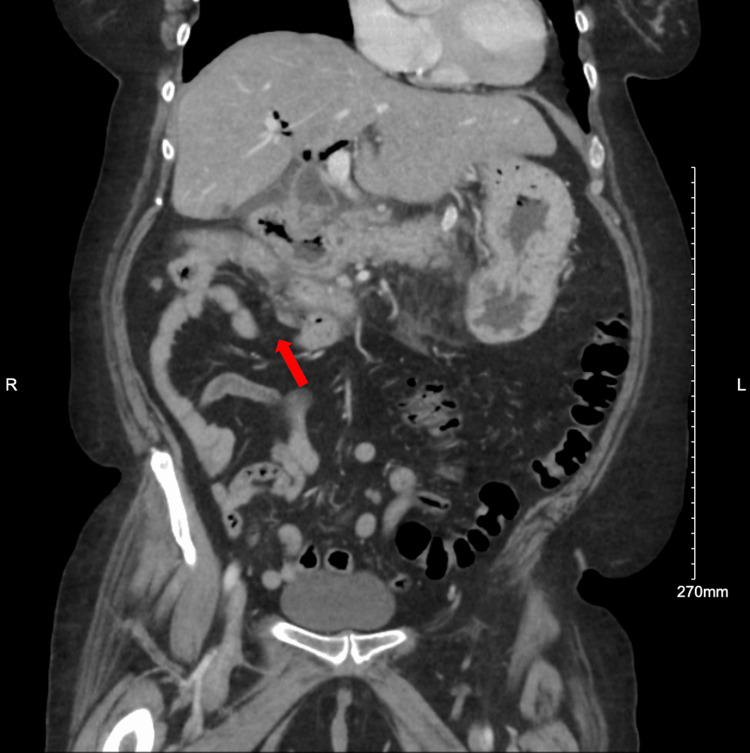
Coronal computed tomography (CT) abdomen/pelvis with contrast. Arrow points to a malrotated duodenum in the right upper quadrant of the abdomen.

A 61-year-old female with a reported history of cholecystectomy, type 2 diabetes, and alcohol use disorder presented to the emergency department with two weeks of right-sided abdominal pain, nausea, and anorexia. She was hypertensive with right upper quadrant tenderness on physical exam. Her lab studies (Table 1) revealed elevated alkaline phosphatase (147 U/L) with otherwise normal transaminases, elevated lipase (1620 U/L), total hyperbilirubinemia (1.6 mg/dL), leukocytosis with a left shift (WBC 17.9/L), hyperglycemia (596 mg/dL), high anion gap metabolic acidosis (venous pH 7.14, CO2 7 mmol/L, anion gap 31 mmol/L. A computed tomography (CT) scan of her abdomen with contrast (Figures 1-2) showed abnormal appearing thick-walled proximal small bowel and a malrotated duodenum with surrounding inflammatory changes extending to the pancreas suspicious of fistulous communication to the bile ducts where there was extensive pneumobilia. She received fluids, ceftriaxone, metronidazole, and insulin and was transferred to our hospital.

**Table 1:** Blood test results from initial emergency department visit. Glucose, blood urea nitrogen, anion gap, alkaline phosphatase, lipase, total bilirubin, white blood cell count, absolute neutrophil count (ANC), and beta-hydroxybutyrate were elevated (H). Sodium, potassium, chloride, CO2, and venous pH were low (L).

Parameters	Value	Reference Range
Glucose	596 mg/dL (H)	70 – 99 mg/dL
Blood urea nitrogen	47 mg/dL (H)	8 – 23 mg/dL
Creatinine	0.94 mg/dL	0.60 – 1.10 mg/dL
Sodium	118 mmol/L (L)	135 – 145 mmol/L
Potassium	3.3 mmol/L (L)	3.5 – 5.3 mmol/L
Chloride	80 mmol/L (L)	98 – 108 mmol/L
CO2	7 mmol/L (L)	23 – 32 mmol/L
Anion gap	31 mmol/L (H)	7 – 16 mmol/L
Aspartate aminotransferase	10 U/L	0 – 37 U/L
Alanine aminotransferase	15 U/L	6 – 37 U/L
Alkaline phosphatase	147 U/L (H)	35 – 117 U/L
Lipase	1620 U/L (H)	16 – 63 U/L
Bilirubin, total	1.6 mg/dL (H)	0.0 – 1.2 mg/dL
White blood cell	17.1 10*9/L (H)	4.0 – 11.0 10*9/L
Neutrophils (ANC) absolute	14.8 10*9/L (H)	1.1 – 6.1 10*9/L
Hemoglobin	15.1 g/dL	12 – 16 g/dL
Platelets	194 10*9/L	140 – 440 10*9/L
pH, Venous	7.14 (L)	7.32 – 7.42
Beta-hydroxybutyrate	8.38 mmol/L (H)	0.02 – 0.27 mmol/L

**Figure 3:**
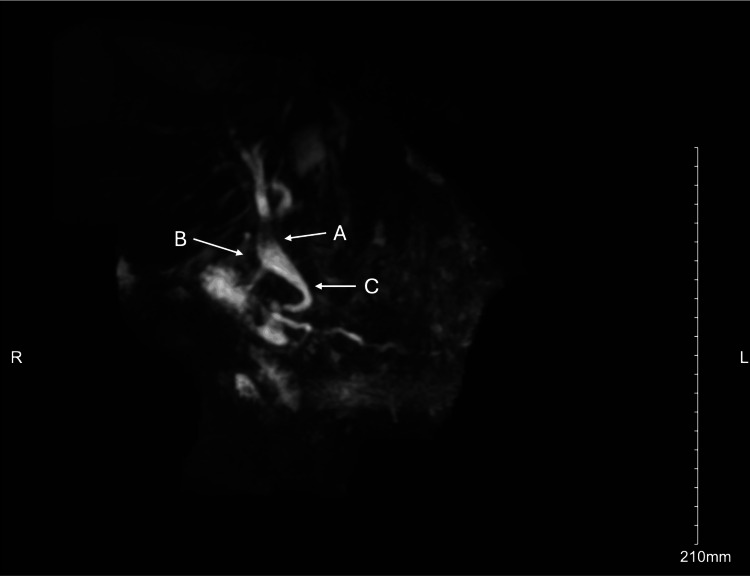
Magnetic resonance cholangiopancreatography (MRCP) image findings. The image demonstrates persistent extrahepatic biliary duct dilation with the fistulous tract from the common bile duct (CBD) to the proximal duodenum. (A: proximal CBD, B: fistulous connection, C: distal and obstructed CBD).

Her complete medical record was obtained following her arrival at our facility and revealed that she had undergone an open cholecystectomy and side-to-side choledochoduodenostomy two years prior. Magnetic resonance cholangiopancreatography (MRCP) demonstrated persistent extrahepatic biliary ductal dilation with a fistulous tract from the proximal CBD to the proximal duodenum and edema along the pancreatic bed, suggesting pancreatitis (Figure 3). Endoscopic retrograde cholangiopancreatography (ERCP) revealed an opening at the second part of the duodenum, appearing classic for a functioning choleduodenal anastomosis. The ampulla of Vater was consistent with a prior sphincterotomy; the CBD was cannulated without stenting, and fibrous food debris was extracted until no evidence of filling defects remained. The findings were consistent with the sump syndrome. The patient’s post-operative course was unremarkable, and she was discharged home in stable condition.

## Discussion

**Figure 4:**
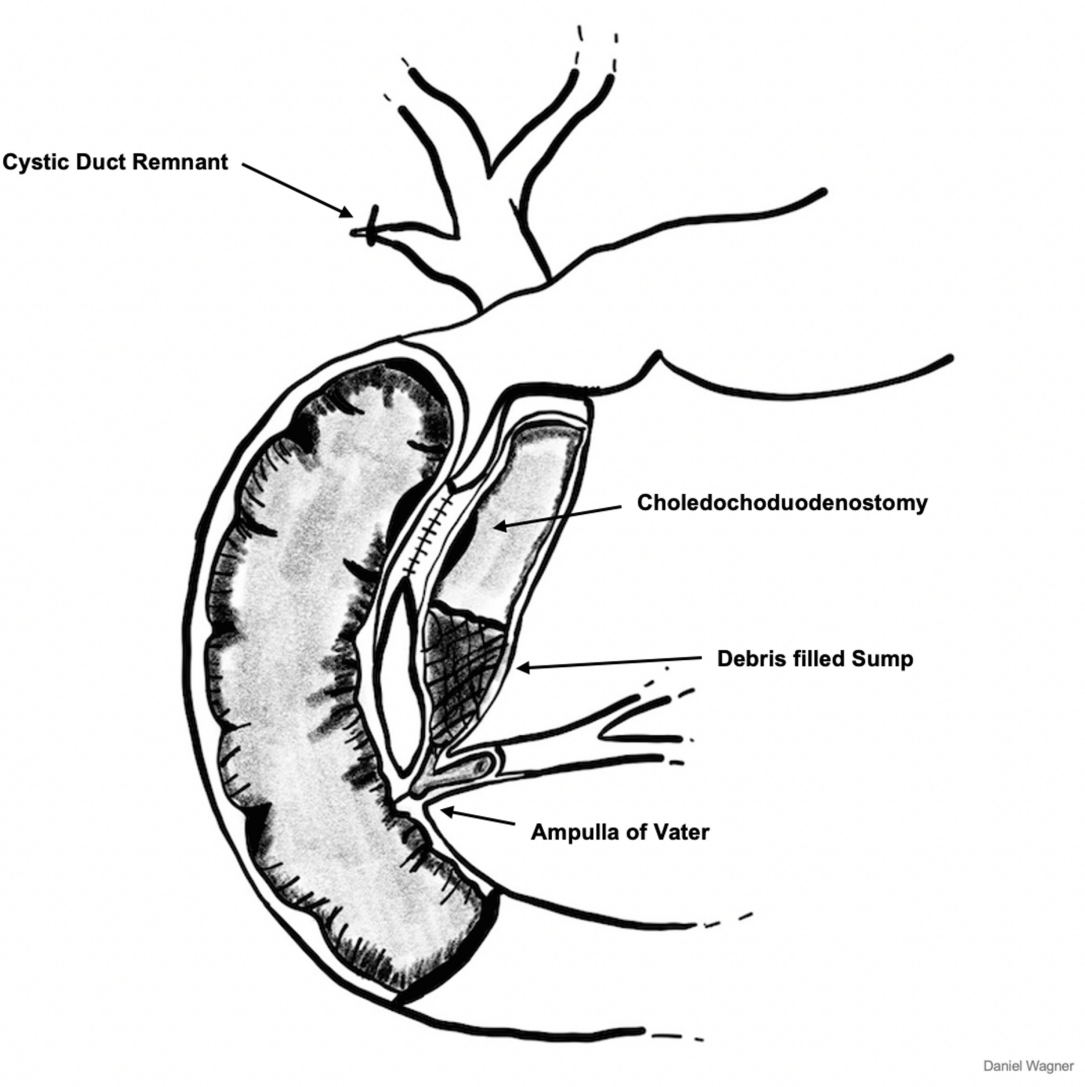
Schematic representation of a choledochoduodenoanastomosis and “sump” formation. Image credit: Daniel Wagner, who gave permission to publish with this article.

A sump is a pit or reservoir serving as a liquid drain or receptacle [5]. In sump syndrome, a sump is formed as a complication of biliary bypass surgery, most commonly CDD, with an average timeframe to appear of 6 - 11 years postoperatively [6-12]. The resulting anatomy after a CDD creates a potential space for sump syndrome to develop (Figure 4). The incidence of sump syndrome following a CDD is variable, with reported values ranging from 0 - 15.7% [13,14]. This variation can be attributed partly to the length of time to presentation and the lack of a precise definition of the syndrome. Our patient presented two years after her CDD, making it an unusually early occurring case of sump syndrome.

A combination of clinical and radiologic findings best characterizes the sump syndrome. Though not always present, clinical symptoms are often recurrent and include right upper quadrant pain, jaundice, fever, liver abscesses, pancreatitis, and signs of cholangitis. Ultrasound, CT, and MRCP are all imaging modalities that can suggest sump syndrome. Radiological signs of suspicion include debris or gallstones in the distal CBD and pneumobilia [8]. Pneumobilia may indicate a functional anastomosis in a patient with an unknown surgical history. In a patient with a known history of CDD, this may be a normal finding. However, these radiographic findings warrant further investigation in conjunction with clinical symptoms or in a patient without detailed history. 

Despite clinical signs and symptoms indicative of sump syndrome, the diagnosis is confirmed with ERCP. Diagnosis and treatment may occur concomitantly because ERCP with sphincterotomy of the ampulla of Vater should be the first-line treatment in most instances of the syndrome [10,14,15]. In a large retrospective cohort study of 70 patients with a history of CDD, Demirel et al. found that endoscopic sphincterotomy successfully diagnosed and definitively treated all 11 with sump syndrome [14]. However, definitive surgery may be performed if endoscopic management fails or repeat intervention is required [6,16]. Recurrence of the syndrome after endoscopic sphincterotomy is not uncommon, as indicated by Mavrogiannis et al. In a case series of 31 patients, nearly 20% experienced restenosis of the sphincterotomy opening [15]. Surgical options are limited but include conversion to other biliary diversion options such as hepaticojejunostomy or distal gastrectomy with Roux-en-Y gastrojejunostomy [6,8].

## Conclusions

It is essential to consider sump syndrome in patients with cholangitis or pancreatitis who have a history of biliary surgical intervention, especially when their procedural details are unknown. The case presented affirms MRCP as a valuable diagnostic tool for sump syndrome and is notable as an early-occurring example of a typically late-presenting complication of CDD. Identifying the diagnosis of sump syndrome is critical for definitive treatment and avoiding future episodes of potentially life-threatening complications like cholangitis.
